# Desipramine treatment disrupts phagocytic and intracellular survival of *Brucella abortus* 544 in RAW 264.7 cells and promotes bacterial resistance with enhanced immune responses in ICR mice

**DOI:** 10.3389/fvets.2026.1779164

**Published:** 2026-05-13

**Authors:** Tran Xuan Ngoc Huy, Said Abdi Salad, Trang Thi Nguyen, Ched Nicole Turbela Aguilar, Eoin Quiatchon Lajara, Wongi Min, Hu Jang Lee, Alisha Wehdnesday Bernardo Reyes, Suk Kim

**Affiliations:** 1 HUTECH Institute of Applied Sciences, HUTECH University, Ho Chi Minh City, Vietnam; 2 Institute of Animal Medicine, College of Veterinary Medicine, Gyeongsang National University, Jinju, Republic of Korea; 3 Department of Veterinary Paraclinical Sciences, College of Veterinary Medicine, University of the Philippines Los Baños, College, Laguna, Philippines

**Keywords:** *Brucella abortus*, desipramine, ICR mouse, internalization, intracellular growth, pro-inflammatory cytokines, RAW 264.7 cells

## Abstract

**Introduction:**

*Brucella (B.)* abortus is an intracellular pathogen that causes chronic infection and remains difficult to treat. In this study, desipramine (DMI) was investigated as a potential host-directed therapeutic strategy to control *Brucella* infection.

**Methods:**

The effects of DMI on B. abortus infection were evaluated using RAW 264.7 macrophages and an in vivo ICR mouse model. Bacterial growth, uptake, and intracellular survival were assessed in macrophages. Gene expression and signaling pathway analyses were performed to investigate immunomodulatory mechanisms. Infected-mice were treated with DMI, and bacterial loads in target organs, as well as immune responses, were examined.

**Results:**

DMI exhibited a direct inhibitory effect against *Brucella* growth as well as on the bacterial uptake and intracellular survival within macrophages. This effect was accompanied by increased expression of genes that are known to play crucial roles in the control of *Brucella* infection. Furthermore, DMI treatment resulted in the suppression of phospho-ERK1/2, MALT1 and Bcl10 protein expression, suggesting that the disruption of Brucella phagocytosis trafficking involves downstream signaling pathways regulated by these proteins. Notably, DMI treatment in *Brucella*-infected mice significantly reduced bacterial loads in the spleen and showed a decreasing trend in the liver, along with enhanced Th1-associated immune responses.

**Conclusion:**

Together, these findings indicate that DMI exhibits both bactericidal and immunomodulatory effects beneficial to the prevention, treatment, and control of *Brucella* infection.

## 1 Introduction

Brucellosis remains a persistent, re-emerging, and neglected zoonotic infectious disease that presents major challenges to public health and substantial socio-economic burden, contributing to more than 2.1 million new cases annually (1). The etiologic agent, *Brucella,* is a small Gram-negative, facultative intracellular coccobacillus that lacks spores, flagella, and capsules, yet has a broad host range. Domestic animals such as cattle, goats, and sheep are among its most common hosts. Humans are also susceptible to *Brucella* infection (1, 2). The core species of the genus include *Brucella* (*B.*) *abortus, B. canis, B. melitensis, B. neotomae, B. ovis,* and *B. suis*, which are distinguished by their specific host preferences (2). In animals, the pathogen causes epididymitis and orchitis in males, and abortions, stillbirths, premature births, and postpartum metritis in females (3, 4). Humans primarily acquire brucellosis through direct contact with secretions from the infected female animals, and also via the consumption of unpasteurized dairy products and contaminated food and water. Although rare, inhalation of *Brucella-*containing aerosols is another possible means of infection (4). The clinical manifestations of human brucellosis are non-specific and can lead to misdiagnosis or delayed diagnosis (5). The early symptoms include fever, general fatigue, joint pain, and night sweats, which, if left untreated, may progress to complications involving the cardiovascular or nervous systems and even be life-threatening in severe cases (6).

The non-specific nature of brucellosis necessitates a thorough clinical evaluation, along with appropriate serological testing. Serological assays such as the Rose Bengal test, standard tube agglutination test (STAT), and enzyme-linked immunosorbent assay (ELISA) are most commonly employed to detect *Brucella*-specific antibodies in serum. However, cross-reactivity with other bacterial species and variability in test sensitivity and specificity complicate the diagnostic process (5, 7). In addition to limited surveillance, brucellosis remains underreported in many regions despite global control efforts involving animal vaccination and public health interventions, with its true incidence estimated to be 10–25 times higher than reported, according to the World Health Organization (5, 6).

The treatment of human brucellosis remains challenging due to the need for prolonged administration of multiple antibiotics and their associated adverse side effects (8, 9). With the growing problem of antibiotic resistance in microorganisms, which is also a global health concern, further advances have focused on developing alternative, non-antibiotic therapeutic strategies. Notably, the spread of antibiotic resistance is reported to be responsible for over 700,000 deaths worldwide each year, and this number is projected to rise dramatically in the future (10). Previous studies reported the potential antibacterial effect of certain antidepressants, such as the use of imipramine against *Brucella* (11, 12). In this study, we investigated the potential inhibitory effects of desipramine (DMI), a major active imipramine metabolite and a tricyclic antidepressant, against *Brucella* pathogenesis using both *in vitro* and *in vivo* models, with the aim of finding alternative treatment against brucellosis.

## 2 Materials and methods

### 2.1 Reagents

Desipramine (DMI, catalog no. D3900; molecular weight, 266.381 g/mol) was purchased from Sigma-Aldrich (MO, United States) and dissolved in triple-distilled water (DW) to prepare a stock solution of 100 mM concentration. The stock solution was sterilized by filtration through a 0.2 μm pore-sized membrane, stored at −20 °C, and further diluted in phosphate-buffered saline (PBS). Fetal bovine serum (FBS) and 1% antibiotic-antimycotic (AA) solution were purchased from Thermo Fisher Scientific, Inc. (NY, United States). 3-(4,5-dimethylthiazol-2-yl)-2,5-diphenyltetrazolium bromide (MTT) and gentamicin were purchased from Sigma-Aldrich (MO, United States). The BD cytometric bead array (CBA) Mouse Inflammation Kit was from BD Biosciences (CA, United States), Brucella broth from Becton Dickinson (NJ, United States), and agar was from Yakuri Pure Chemicals Co., Ltd. (Kyoto, Japan). The RNeasy Plus Mini Kit and QuantiTect^®^ Reverse Transcription Kit were purchased from Qiagen (Hilden, Germany), red blood cell (RBC) lysis buffer was purchased from Roche (Basel, Switzerland), and radioimmunoprecipitation assay (RIPA) buffer was purchased from Thermo Fisher Scientific, Inc. (NY, United States). The mouse IgG2a Uncoated ELISA Kit with Plates was purchased from Invitrogen (Vienna, Austria). Primary rabbit polyclonal anti-MALT1, anti-Bcl10, anti-β-actin, anti-pan-ERK1/2, and anti-phospho-ERK1/2 antibodies were purchased from Cell Signaling Technology, Inc. (MA, United States), and FITC-conjugated rat anti-mouse CD4^+^ and PE-conjugated rat anti-mouse CD8^+^ monoclonal antibodies were purchased from Invitrogen (Vienna, Austria).

### 2.2 Macrophage and bacteria

Murine RAW 264.7 cells (TIB-71; VA, United States) were maintained in 25- or 75-cm^2^ culture flasks with RPMI 1640 medium (Thermo Fisher Scientific, Inc., NY, USA) supplemented with 10% heat-inactivated FBS and 1% AA solution. Cells were incubated in 5% CO_2_ atmosphere at 37 °C prior to all infection assays, the concentration of cells used per well was 7 × 10^4^, and the cell medium was changed to fresh medium without AA.

*Brucella abortus* 544 biovar 1 (ATCC 23448) was kindly provided by the Laboratory of Bacteriology Division of the Animal and Plant Quarantine Agency in the Republic of Korea. The bacteria were cultivated on Brucella agar (1.5% w/v) for 2 days at 37 °C, and one colony was then aseptically cultured into 5 mL of Brucella broth for 2 days in a shaking incubator (37 °C, 180 rpm). Serial dilutions were plated on agar plates, and the bacterial colonies were counted to determine bacterial concentration. Biosafety level 3 practices were performed in handling bacteria.

### 2.3 MTT assay

RAW 264.7 cells were cultured overnight in a 96-well plate and incubated with different concentrations of DMI (0, 0.01, 0.02, 0.04, 0.06, 0.08, 0.10, 0.20, 0.50, and 1.00 mM) for 48 h in a 5% CO_2_ atmosphere at 37 °C. PBS was used as a control since it is the diluent used to dissolve DMI. After incubation, the cells were washed, and the medium was replaced with fresh culture medium containing MTT solution (5 mg/mL). The plates were incubated for an additional 3 h, and the medium was replaced with 150 μL of dimethyl sulfoxide (DMSO). After 15 min, the absorbance in each well was measured at 540 nm using a microplate spectrophotometer reader (Multiskan Sky; Thermo Scientific, MA, United States). The highest DMI concentration that did not significantly affect the cell viability was used in the succeeding experiments with RAW 264.7 cells.

### 2.4 Bactericidal assay

*Brucella* cultured in broth were diluted in PBS to have a final concentration of 1 × 10^5^ colony-forming units (CFUs) per well in a 96-well U-bottom plate. The direct antibacterial effect of DMI was evaluated by incubating the bacteria with different concentrations of DMI (0, 0.1, 1, 10, and 100 mM) in PBS and (0, 0.08, 0.8, 8, and 80 mM) in RPMI for 5 min, 5 h, 24 h, and 48 h at 37 °C. Following incubation, samples from each well were serially diluted in PBS and plated on agar plates. After 3 days of incubation, bacterial colonies were counted, and results were expressed as log_10_ CFU counts.

### 2.5 Cell infection assays

The treatment groups consist of two different concentrations of DMI (0.04 and 0.08 mM) in a 96-well plate. For the evaluation of internalization efficiency of *Brucella,* RAW 264.7 cells were prepared as that of the MTT assay and pre-incubated with DMI for 0.5 h. The cells were then washed prior to bacterial infection to prevent the potential direct effect of DMI with the bacteria. The cell medium was changed to fresh medium without antibiotics and then infected with *Brucella* at a multiplicity of infection (MOI) of 200. The plate was centrifuged at 150 × *g* for 10 min at room temperature to promote bacteria-to-cell interaction. This was then followed by incubation for 5, 20, and 60 min at 37 °C prior to washing once with PBS, and further incubated for 30 min with fresh medium containing 100 μg/mL gentamicin to eliminate extracellular bacteria. Finally, the cells were washed twice with PBS, lysed in DW, and serially diluted before plating on agar plates to count bacterial colonies.

To evaluate the intracellular survival efficiency of *Brucella,* RAW 264.7 cells were prepared following the same procedure as in the internalization assay, but the cells were infected first with *Brucella* at an MOI of 200 for 1 h at 37 °C. After infection, the cells were washed with PBS and then treated with fresh medium containing different concentrations of DMI and 100 μg/mL gentamicin. The plates were further incubated for 2, 24, and 48 h at 37 °C. At the end of each incubation period, the cells were washed twice with PBS, lysed in DW, and serially diluted before plating on agar plates to count bacterial colonies. The CFUs were expressed in log_10_.

### 2.6 RNA isolation and quantitative real-time-PCR

RAW 264.7 cells were prepared in a 6-well plate, and the protocols for internalization or intracellular survival assays were performed for 5 min and 48 h, respectively. The total RNA was extracted using the RNeasy Plus Mini Kit and reverse-transcribed into cDNA using QuantiTect^®^ Reverse Transcription Kit, following the manufacturer’s instructions. qRT-PCR amplification reactions were carried out using the thermal cycling conditions as described by Huy et al., and the oligonucleotide primer sequences are listed in Table 1 (13). qRT-PCR analysis was done as previously described (13, 14).

**Table 1:** Primer sequences used for quantitative real-time PCR.

Gene	Common name	Forward (5′–3′)	Reverse (5′–3′)
*Gapdh*	Glyceraldehyde-3-phosphate dehydrogenase	GGA GAA ACC TGC CAA GTA TG	AAC CTG GTC CTC AGT GTA G
*Cdc42*	Cell division cycle 42	AAA AGT GGA TAC AAA ACT GTT TCC	CTC TGG AGT AAT AGG CTT CTG TTT GT
*rhoA*	Ras homolog gene family member A	ACT CGG AGT CCT CGC CTT GA	TCT GGG AAC TGG TCC TTG CTG
*Rac1*	Rac family small GTPase 1	GTC CCA ATA CTC CTA TCA TCC TCG	GAG CAC TCC AGG TAT TTG ACA GCA
*SYK*	Spleen tyrosine kinase	TCC ATG GCA ACA TCT CCA G	GAC ATG GTA CCG TGA GGA
*TLR2*	Toll-like receptor 2	CTG AGA ATG ATG TGG GCG TG	ATG GGA ATC CTG CTC ACT GTA G
*TLR4*	Toll-like receptor 4	AGT TTA GAG AAT CTG GTG GCT GTG	TTC CCT GAA AGG CTT GGT CT
*IL1β*	Interleukin-1 beta	GGC AGG CAG TAT CAC TCA TTG TGG	CTC ATG TCC TCA TCC TGG AAG G
*IFNβ*	Interferon beta 1	GTA CGT CTC CTG GAT GAA CTC C	CAA GTG GAG AGC AGT TGA GG
*IL6*	Interleukin-6	ACC ACG GCC TTC CCT ACT T	CAT TTC CAC GAT TTC CCA GA
*IL10*	Interleukin-10	GGG TTG CCA AGC CTT ATC GG	CTC TTC ACC TGC TCC ACT GC
*MCP1*	Monocyte chemoattractant protein-1	CCC AAT GAG TAG GCT GGA GA	AAA ATG GAT CCA CAC CTT GC
*TNFα*	Tumor necrosis factor alpha	CAG GTT CTG TCC CTT TCA CTC ACT	GTT CAG TAG ACA GAA GAG CGT GGT
*iNOS2*	Inducible Nitric oxide synthase 2	GGA GGT GCT TGA AGA GTT CC	AGG AGC TGA TGG AGT AGT AGC

### 2.7 Western blotting

The same preparation of RAW 264.7 cells was done as that of the qRT-PCR experiment, and the infection and treatment of cells were conducted as in the internalization and intracellular survival assay. The cell lysates were collected at 5 min and 48 h post-infection (pi) using ice-cold RIPA buffer with 1% protease inhibitor cocktail and incubated for 20 min at 4 °C. Protein concentrations were then measured using the Bradford protein assay. Equal amounts of protein extracts (22 μg per well) were separated by 12% sodium dodecyl sulfate-polyacrylamide gel electrophoresis (SDS-PAGE) gels, and transferred onto nitrocellulose membranes for 20 min, and underwent immunoblotting with the indicated antibodies. Primary rabbit polyclonal anti-MALT1, anti-Bcl10, anti-β-actin, anti-pan-ERK1/2, and anti-phospho-ERK1/2 antibodies were diluted in Tris-buffered saline with 0.1% Tween 20 containing 5% skim milk, followed by incubation with secondary goat anti-rabbit HRP-conjugated antibody. Images were acquired using a Molecular Imager^®^ ChemiDoc™ XRS + system (Bio-Rad Laboratories, CA, United States), and the signal intensities were analyzed using Image Lab™ software version 5.0.

### 2.8 Protective efficacy analysis in mice

Handling and all the experimental protocols performed with the animals were in accordance with the guidelines of the Animal Ethical Committee of Chonbuk National University, approved under Authorization Number CBNU-2021-037. Eight-week-old specific pathogen-free female ICR mice were purchased from Samtako Bio Co. Ltd. (Osan, Republic of Korea) and were randomly grouped into non-infected and *Brucella-*infected groups. These were further subdivided into non-treated and DMI-treated groups of 6 mice each. Animals were housed in metabolic cages under standard laboratory environment conditions, maintained on a standard 12 h light/12 h dark cycle, and acclimatized for 1 week with provision of *ad libitum* distilled drinking water and food.

**Figure 1:**
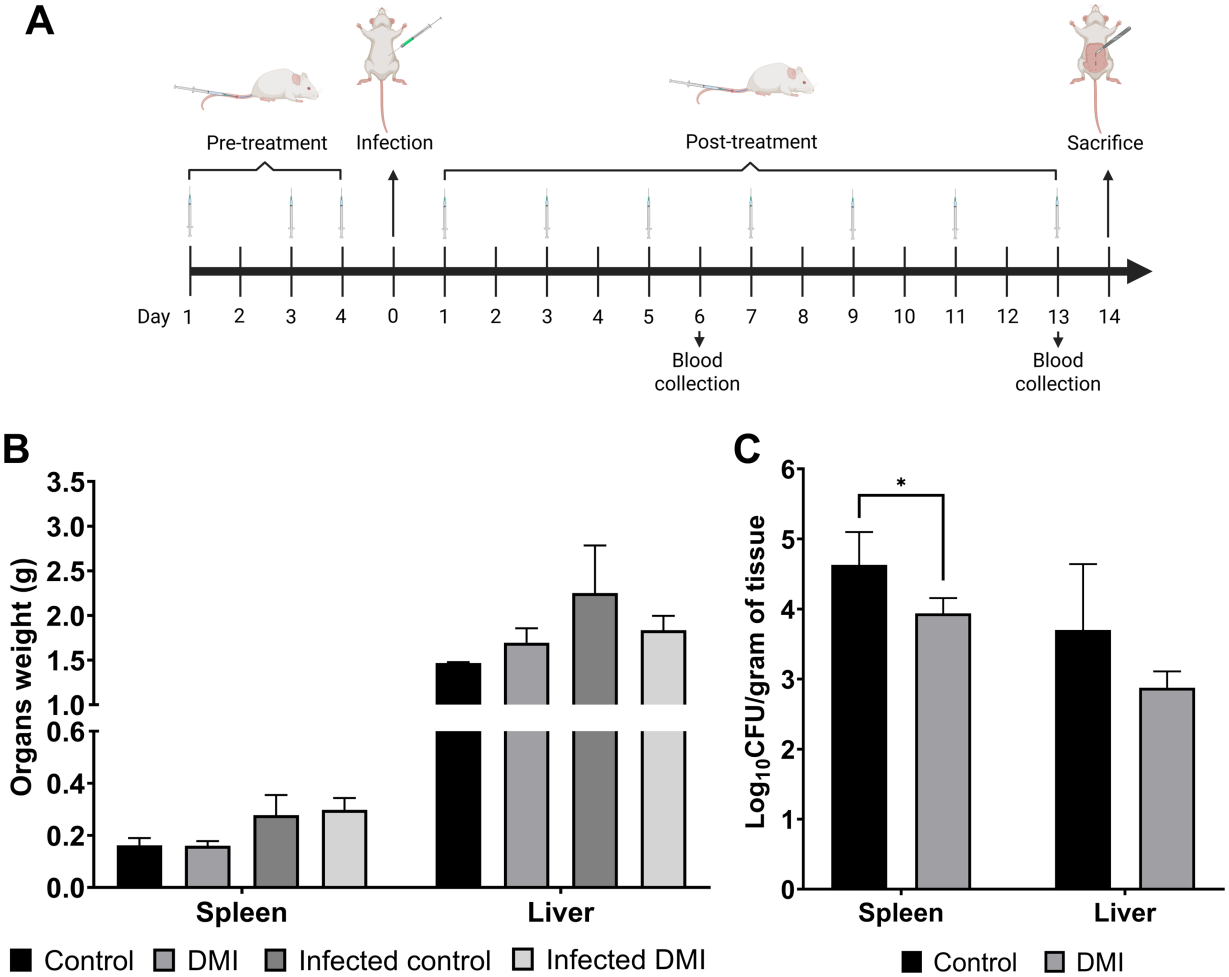
Effect of desipramine on *B. abortus* persistence in ICR mice. Following the experiment design **(A)**. The average weight of spleens and livers of desipramine-treated mice was evaluated **(B)**, and the bacterial proliferation in these organs was expressed in log_10_ CFU **(C)**. The data are expressed as the mean ± SD of each group of 6 mice. *p < 0.05; **p < 0.01; ***p < 0.001.

One group from both the non-infected and *Brucella-*infected groups received intravenous injection via the tail vein of either 100 μL of PBS or 100 μL of DMI (10 mg/kg/day per mouse, filtered in a 0.45 μm pore-size membrane) on days 1, 3, and 4 prior to infection with *Brucella* for the infected groups on day 5. This pre-treatment is for evaluation of the effect of DMI for its potential preventive efficacy against *Brucella* infection. The bacterial concentration used was 1 × 10^6^ CFUs in 100 μL PBS and inoculated via the intraperitoneal route in each animal. DMI administration was continued after 24 h of infection every other day until 14 days pi. Blood samples were collected via the tail vein on days 6 and 13 pi for measurement of CD4^+^ and CD8^+^ T cell populations, serum IgG2 titers, and cytokine levels. During the entire experimental period, mice were monitored for clinical symptoms. The animals were sacrificed at 14 days pi via cervical dislocation, and the liver and spleen were aseptically collected and weighed (Figure 1A). A 0.05 g portion of each organ was collected and homogenized in PBS to determine bacterial proliferation. The homogenized organs were serially diluted in PBS, plated on agar plates, and incubated for 3 days at 37 °C. The bacterial colonies were counted and expressed in log_10_ to calculate protection units.

### 2.9 Flow cytometry

Blood samples were centrifuged at 2,000 × *g* for 10 min at 4 °C, and serum was carefully collected. Fifty microliters of each serum sample were processed following the manufacturer’s instructions for quantifying the levels of IL12p70, TNFα, IFNγ, MCP1, IL10, and IL6 using BD CBA Mouse Inflammation Kit. Separately, cellular populations in peripheral blood were examined at the indicated time points, and 70 μL of the collected blood was added to 75 μL of FITC-conjugated anti-mouse CD4^+^ and PE-conjugated anti-mouse CD8^+^ monoclonal antibodies. After staining and incubation in the dark, the mixtures were treated with RBC lysis buffer, and the reaction was terminated using PBS.

The acquisition of data was performed using a FACSLyric™ flow cytometer (BD Biosciences, CA, United States) and analyzed using BD FACSuite software. The data on the cytokine were analyzed by the BD company to determine the concentration at the indicated time points.

### 2.10 Elisa

Serum samples were processed in accordance with the manufacturer’s instructions for quantifying mouse IgG2a. Briefly, the resulting coated plate was added with 50 μL of serum, followed by addition of the diluted Detection Antibody provided in the kit. After incubation and washing, 100 μL of Substrate Solution was added to each well, and the plate was further incubated at room temperature for 15 min. The reaction was terminated using 100 μL of Stop Solution, and the absorbance was measured at 450 nm using a microplate reader.

### 2.11 Statistical analysis

All the *in vitro* experiments were independently repeated at least three times with at least three replicates while the *in vivo* experiments involved six animals per group. The data were analyzed using an unpaired Student’s *t-*test and two-way ANOVA. The graphs were generated using GraphPad Prism. The results were expressed as mean ± standard deviation (SD), and the differences with *p* < 0.05 were considered statistically significant.

## 3 Results

### 3.1 Effect of desipramine treatment on the survivability of macrophages and *Brucella*

**Figure 2:**
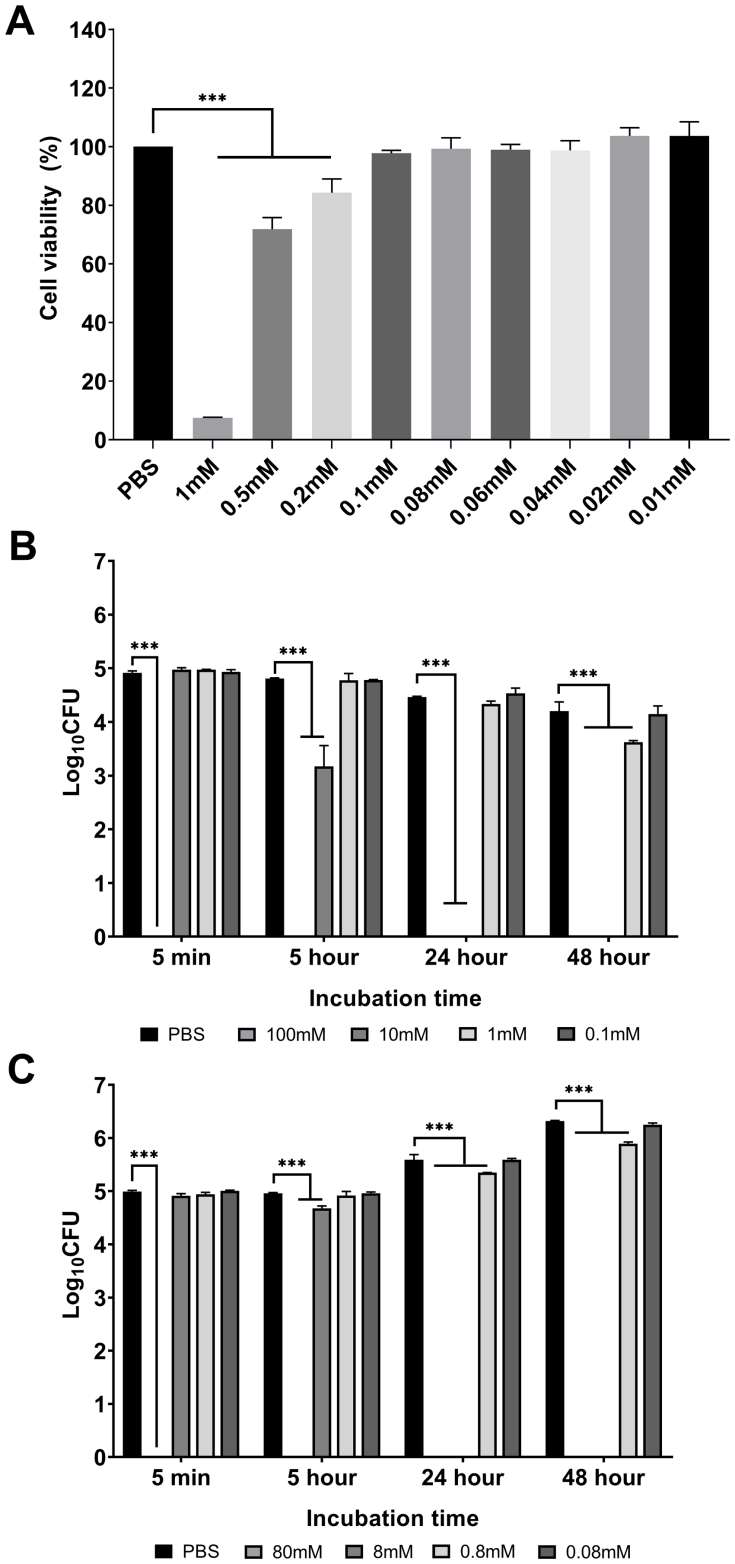
Effect of desipramine on RAW 264.7 cells and *B. abortus* growth. RAW 264.7 cells were incubated with various concentrations of desipramine, and the cell viability was assessed and expressed as a percentage **(A)**. The direct effect of desipramine on *B. abortus* growth in PBS **(B)** and in RPMI 1640 **(C)** was evaluated and expressed in log_10_ CFU. The data are expressed as the mean ± SD of at least three samples. *p < 0.05; **p < 0.01; ***p < 0.001.

The highest non-cytotoxic concentration of DMI that did not significantly affect the viability of RAW 264.7 cells was 0.08 mM (99.27 ± 3.76%, *p* > 0.999) (Figure 2A). This concentration was used in subsequent *in vitro* assays with macrophages.

The direct antibacterial effect of DMI was also investigated by incubating bacteria with varying concentrations of DMI in PBS (Figure 2B), showing a bactericidal effect against *Brucella* at 5 min of incubation at a concentration of 100 mM. At 5 h of incubation, DMI at 10 mM (3.18 ± 0.38 log10 CFU, *p <* 0.001) significantly inhibited the growth of bacteria. Furthermore, at the same concentration, no growth was observed at 24 h of incubation. The concentration of DMI at 1 mM (3.62 ± 0.03 log10 CFU, *p <* 0.001) was observed to significantly reduce the bacterial growth at 48 h of incubation, whereas the lowest concentration tested (0.1 mM) did not affect bacterial growth at any time point.

When using RPMI 1640 medium, the highest DMI concentration that did not affect RAW 264.7 cell viability was used as the minimum concentration to test its direct effect on *Brucella* growth. This concentration exhibited no bactericidal activity at any of the time points examined. However, higher concentrations (0.8, 8, and 80 mM) demonstrated bactericidal effects, particularly with prolonged incubation (Figure 2C). Although bacterial growth was relatively enhanced in RPMI 1640, likely due to the nutrient composition of the medium, increasing DMI concentrations (10-1,000-fold) suppressed bacterial proliferation, showing a reduction pattern similar to that observed in PBS.

### 3.2 Effect of desipramine treatment on the uptake and intracellular survival of *Brucella abortus* in macrophages

**Figure 3:**
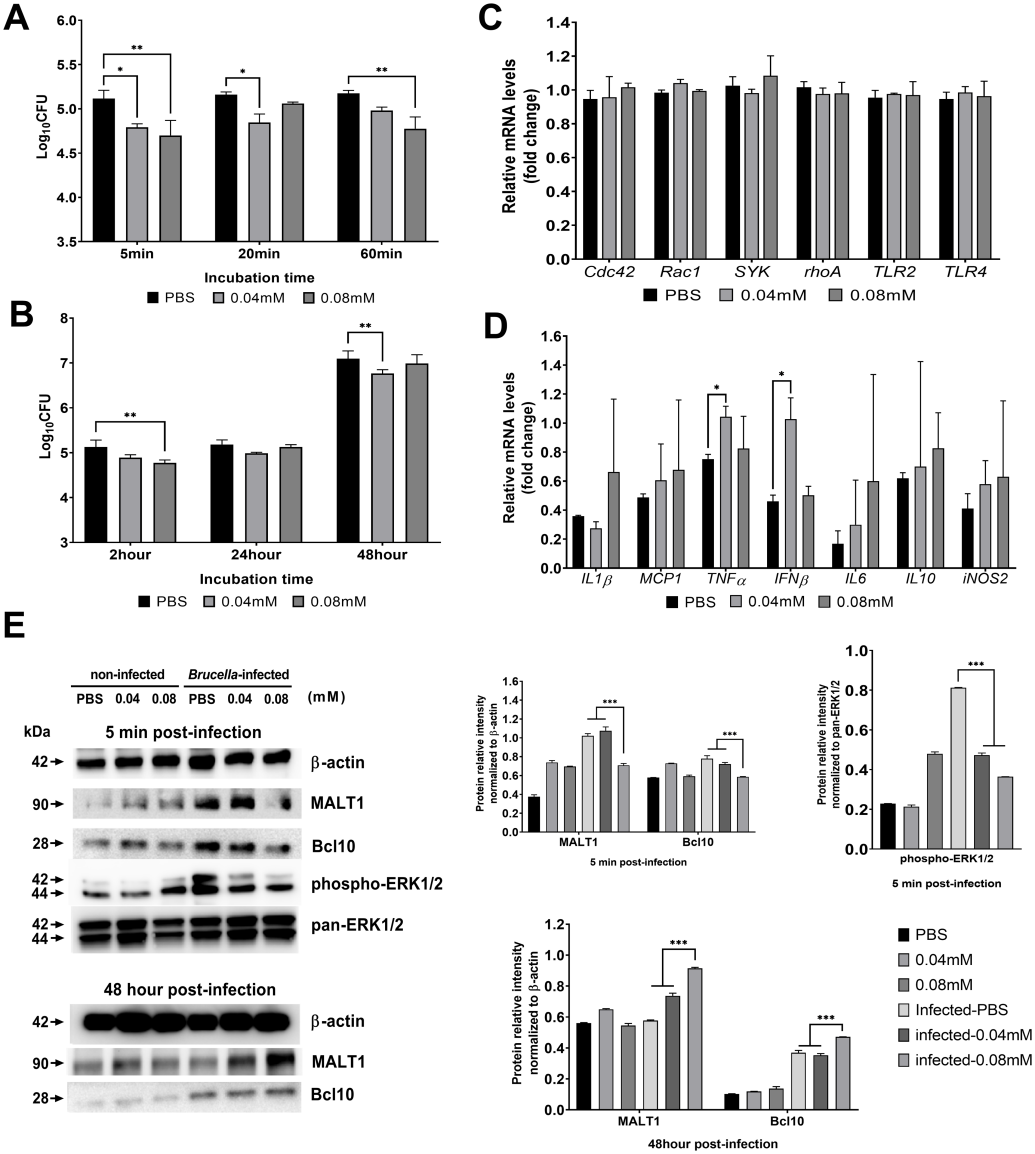
Effect of desipramine on the internalization and intracellular survival of *B. abortus* in RAW 264.7 cells. Internalization efficiency **(A)**, intracellular survival efficiency **(B)**, transcriptional profiles of genes involved in immune signaling and cytoskeletal regulation **(C,D)**, and intracellular signaling assessed using antibodies against MALT1, Bcl10, β-actin, pan-ERK1/2, and phospho-ERK1/2 **(E)**. The data are expressed as the mean ± SD of at least three samples. *p < 0.05; **p < 0.01; ***p < 0.001.

The number of bacteria internalized into macrophages was decreased at concentrations of DMI of 0.04 mM (4.79 ± 0.04 log_10_ CFU, *p <* 0.013) and 0.08 mM (4.70 ± 0.17 log_10_ CFU, *p <* 0.003) at 5 min pi. The reduction was also observed at 60 min pi at the highest concentration used (4.76 ± 0.13 log_10_ CFU, *p <* 0.004) (Figure 3A). DMI-treated cells at 0.08 mM showed reduced intracellular survival of *Brucella* at 2 h post-incubation (4.78 ± 0.06 log_10_ CFU, *p <* 0.004), but not at the later time points tested (Figure 3B). These data suggest that DMI could inhibit both the phagocytosis of *Brucella* into macrophages and intracellular bacterial growth.

On the other hand, the present study investigated the effect of DMI treatment on the expression of several genes involved in the host defense and are targeted and manipulated by *Brucella* in facilitating its entry into macrophages and immune responses evasion or suppression, since at 5 min pi, all the concentrations of DMI that were tested showed reduced bacterial internalization. However, *Cdc42, Rac1, SYK, rhoA, TLR2,* and *TLR4* were not differentially expressed in the cells that were infected with *Brucella* (Figure 3C). In addition, the expression of genes involved in the host’s immune response that are known to affect *Brucella* infection was evaluated at 48 h post-incubation, and the results showed that expressions of *TNFα* and *IFNβ* were augmented particularly in the cells treated at a concentration of 0.04 mM but not with 0.08 mM suggesting that a lower concentration has an immunomodulatory effect during intracellular *Brucella* survival (Figure 3D).

We further examined the effect of DMI treatment on host signaling proteins involved in *Brucella* internalization and intracellular responses. At the early stage of infection (5 min pi), Western blot analysis showed that DMI treatment reduced the expression of MALT1, Bcl10 and ERK1/2 expression. These early changes may contribute to the reduced bacterial internalization observed in DMI-treated macrophages. In contrast, at the later stage of infection (48 h pi), DMI treatment increased the protein expression levels of MALT1 and Bcl10 compared with infected control cells, as confirmed by densitometric analysis normalized to β-actin (Figure 3E). This time-dependent modulation suggests that DMI may differentially regulate host signaling pathways during the early and later stages of *Brucella* infection, potentially influencing both phagocytosis and intracellular growth of *Brucella* inside RAW 264.7 cells.

### 3.3 Effect of desipramine treatment during *Brucella* infection in mice

All groups of mice were examined daily throughout the entire experimental period, and no clinical manifestations of bacterial infection were observed. The groups of mice that were not infected with *Brucella,* showed no differences on the average weight of spleens (0.16 ± 0.02 g) and livers (1.70 ± 0.16 g) as compared to control groups (0.16 ± 0.03 g and 1.46 ± 0.01 g, respectively) (Figure 1B), suggesting that DMI treatment did not significantly induce toxicity, stress or other pathological condition that could lead to the enlargement of organs. The spleen and liver were selected as target organs for evaluating *Brucella* persistence because they are the most affected during infection in mice, particularly the spleen at 2 weeks pi. The groups that were infected with *Brucella* also displayed no significant differences in the average weight of spleens (0.30 ± 0.05 g) and livers (1.83 ± 0.16 g) as compared to control groups (0.28 ± 0.08 g and 2.25 ± 0.53 g, respectively). Interestingly, the number of bacteria per gram in the spleen tissue (3.94 ± 0.07 log_10_ CFU) of the *Brucella-*infected groups was observed to significantly reduce in the DMI-treated groups as compared to control groups (4.63 ± 0.01 log_10_ CFU) while no significant changes were observed in the number of bacteria from the livers of the animals (2.88 ± 0.04 log_10_ CFU) (Figure 1C). The log protection displayed by the DMI-treated mice was 0.69 in spleen. Overall, these findings suggest that DMI treatment may contribute to reduced bacterial persistence and potentially enhance host resistance to *Brucella* infection, although further studies are required to confirm this effect.

### 3.4 Effect of desipramine treatment on the immunity of mice during *Brucella* infection

**Figure 4:**
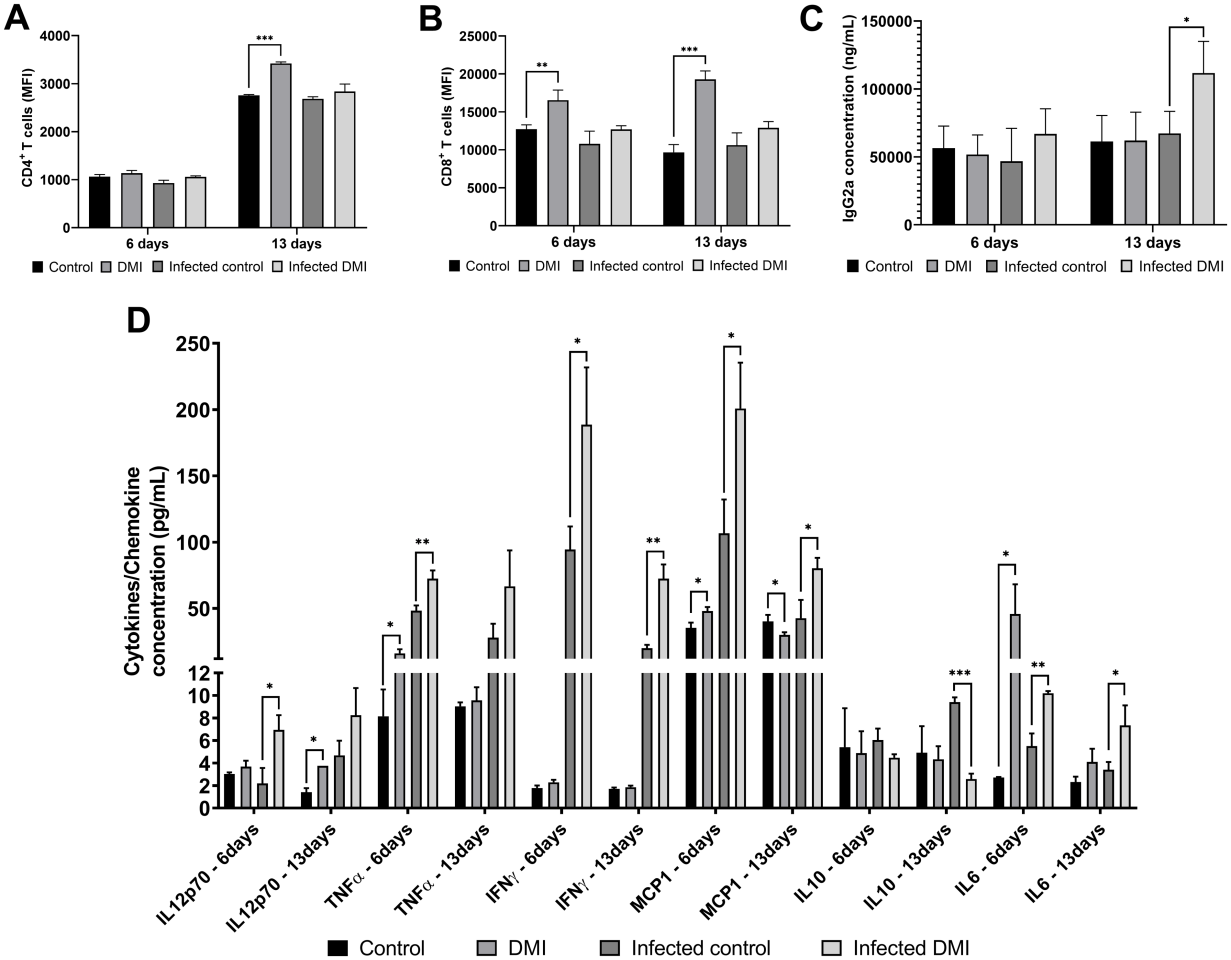
Effect of desipramine on immune responses in ICR mice. CD4^+^ and CD8^+^ mean fluorescence intensities were measured using flow cytometry **(A,B)**, IgG2a antibody production in the sera of mice was measured using ELISA **(C)**, and cytokine concentration in the sera of mice was measured using flow cytometry **(D)**. The data are expressed as the mean ± SD of each group of 6 mice. *p < 0.05; **p < 0.01; ***p < 0.001.

In non-infected mice, the mean fluorescence intensity (MFI) of CD4^+^ in DMI-treated mice was observed to be significantly higher than in the control group at 13 days pi, and a similar pattern was observed in DMI-treated *Brucella*-infected mice at 6 days pi (Figure 4A). The MFI of CD8^+^ from the non-infected groups revealed that without *Brucella* infection, the CD8^+^ levels were observed to be significantly elevated in the treated mice at 6 and 13 days pi; however, CD8^+^ level in the infected groups did not show any significant changes between the treated and the untreated groups, although the values were higher in DMI-treated mice (Figure 4B). These populations of T cells are crucial components of the immune response against intracellular microorganisms such as *Brucella*, and the reduced susceptibility of the animals to *Brucella* infection could be attributed to an increase in the level of CD4^+^ T cells. Moreover, serum IgG2a concentration was observed to significantly increase in the *Brucella-*infected DMI-treated mice at 13 days pi (Figure 4C). This finding indicates that DMI treatment induces a Th1-dominated, cell-mediated immune response, which is essential for controlling intracellular *Brucella.*

Furthermore, it was observed in the present study that the mice treated with DMI displayed induced production of serum level of TNFα (16.14 ± 3.08 pg./mL, *p* = 0.02), MCP1 (47.97 ± 3.05 pg./mL, *p* = 0.012), and IL6 (45.84 ± 22.40 pg./mL, *p* = 0.029) at 6 days pi, and increased IL12p70 (3.76 ± 0.0001 pg./mL, *p* = 0.0114) at 13 days pi in the non-infected groups. For the *Brucella-*infected groups, induced serum levels of IL12p70 (6.95 ± 1.31 pg./mL, *p* = 0.0123), TNFα (72.51 ± 6.19 pg./mL, *p* = 0.0046), IFNγ (188.67 ± 43.24 pg./mL, *p* = 0.0248), IL6 (10.20 ± 0.21 pg./mL, *p* = 0.002), and MCP1 (200.77 ± 34.65 pg./mL, *p* = 0.0193) were observed in the DMI-treated mice at 6 days pi. Among these induced cytokine productions, IFNγ (72.53 ± 10.63 pg./mL, *p* = 0.0011) and IL6 (7.35 ± 1.78 pg./mL, *p* = 0.0231) remained significantly elevated in DMI-treated mice at 13 days pi. In contrast, IL-10 levels (2.58 ± 0.48 pg./mL, *p* < 0.0001) were significantly decreased in mice treated with DMI at 13 days pi (Figure 4D). These results suggested that DMI treatment promotes the production of pro-inflammatory cytokines, which are beneficial for controlling *Brucella* infection.

## 4 Discussion

Brucellosis represents not only a major health constraint on livestock but also a significant threat to public health, resulting in considerable economic losses in many developing countries (15). Since there is currently no vaccine available for humans and the infection can be easily acquired from animals, effective control programs in livestock are critical for eradicating human brucellosis. These programs rely heavily on animal vaccination strategies (16, 17). Although existing vaccines, particularly for the prevention of bovine, caprine, and ovine brucellosis, provide protective immunity, one main problem is the persistent serological responses, aside from the potential for infection to humans and abortion in pregnant cows (16). The global health implications of brucellosis highlight a continuous and urgent need for new strategies to control and eventually eradicate this disease.

*Brucella* species invade and multiply in both professional and non-professional phagocytic cells by manipulation of various host cellular processes (18). Significant causes of human disease are intracellular bacteria that live in host cell-derived vacuoles, such as in the case of *Brucella,* and if left untreated, can result in severe, fatal, or chronic infections leading to extended periods of debilitation (19). These intracellular bacterial pathogens hijack cholesterol metabolism, and thus one of the possible approaches to target different intracellular bacteria is to inhibit acid sphingomyelinase (ASM), which can alter cholesterol trafficking. Desipramine is originally utilized for the treatment of depression, but was employed as a specific inhibitor of ASM activity, which at the same time was also reported to function as a ceramide inhibitor (20). Ceramide, categorized as a sphingolipid, has been reported to play a role in the regulation of bacterial adhesion and invasion (21). In the present study, pretreatment of RAW 264.7 cells with DMI prior to *Brucella* infection reduced bacterial invasion, likely due to ASM inhibition that alters ceramide metabolism. Future studies should further investigate this mechanism to better understand the host-*Brucella* interaction. DMI is a known primary active metabolite of imipramine hydrochloride (ImiP), and in our previous work, the use of ImiP against *B. abortus* 544 showed promising results for its immunomodulatory and antibacterial effects as a potential adjunctive therapy for brucellosis (12). DMI has also been reported to have antimicrobial and fungicidal activities, and suppresses the growth of pathogenic microorganisms; hence emphasizing the concern of the increasing antibiotic resistance, which is becoming a major issue for *Brucella.* However, the findings in this study could lead to other significant approaches, such as targeting ASM or ceramide metabolism for the control of intracellular bacteria, including *Brucella.* While DMI demonstrated bactericidal activity against *B. abortus*, the concentrations used *in vitro* had no direct effect when tested in standard cell culture medium (RPMI 1640), indicating that its inhibitory effect may derive from modulation of ASM, ceramide, or cholesterol metabolism rather than direct antimicrobial action.

*Brucella* employs multiple strategies to manipulate host actin dynamics and endosomal trafficking for efficient internalization and intracellular survival in macrophages. Our data demonstrated that DMI treatment attenuates both the MALT1/Bcl10 and ERK1/2 MAPK signaling pathways, both of which are essential for these processes. *Brucella* infection rapidly induced phosphorylation of ERK1/2 and MALT1/Bcl10 protein levels, consistent with activation of parallel signaling cascades that support NF-κB-mediated inflammatory responses and F-actin remodeling required for bacterial uptake. ERK1/2 signaling, in particular, regulates clathrin-independent endosomal trafficking and promotes actin polymerization at phagocytic cups. DMI significantly reduced phospho-ERK1/2 level and MALT1/Bcl10 expression during infection, thereby impairing these pathways and limiting *Brucella* internalization. Thus, DMI likely disrupts *Brucella* pathogenesis at early internalization stages through coordinated inhibition of ERK1/2-dependent trafficking and MALT1/Bcl10-NF-κB signaling in RAW 264.7 cells (22–24). However, further studies are required to fully elucidate the precise molecular interactions underlying this regulatory mechanism. In this regard, key genes for the control of intracellular bacteria, including *TNFα* and *IFNβ*, were upregulated. This upregulation may enhance protein production beneficial for controlling *Brucella* pathogenesis. The pathogen is known to suppress pro-inflammatory cytokine production to facilitate its survival and evade host immune responses (25). Notably, DMI not only boosted these cytokines but also increased MALT1 and Bcl10 protein levels at 48 h pi (Figure 3E), concomitant with reduced intracellular CFU growth. The MALT1/Bcl10 complex drives NF-κB activation, a central regulator of transcription of *Tnfα* (26). Interestingly, the expression of two of the most important cytokines involved in intracellular bacterial infection was observed to differentially change, and these cytokines are known to play such a major role in the pathogenesis of *Brucella*. TNFα is a crucial cytokine in activating macrophages for effective resistance to *Brucella* infection, and this has been shown to be severely impaired in patients and domestic animals infected with the bacteria. IFNγ is considered the most important protective cytokine against intracellular bacteria, including *Brucella*, as it activates macrophages in order to enhance the ability to kill intracellular pathogens (25, 27, 28). In addition to influencing bacterial adhesion and uptake, DMI displayed intracellular survival inhibitory effect at an early stage of infection, which could be due to the action of DMI in inhibiting ASM resulting to the accumulation of lipids that eventually could result to impairment of lysosome-phagosome fusion and lysosome-associated membrane protein 1 (LAMP1), both of which are important in the control of intracellular *Brucella* in phagocytes (29).

DMI treatment was further evaluated in ICR mice at a dose of 10 mg/kg every other day. Interestingly, the mice that received DMI treatment displayed reduced *Brucella* loads in both the spleen and liver. Although DMI is primarily used as an antidepressant, similar compounds may hold therapeutic potential against a broad range of intracellular bacterial infections. This is particularly relevant given the limited effectiveness of conventional antibiotics, many of which have poor cell membrane permeability. While antibiotics such as tetracyclines can penetrate host cells and are commonly used to treat intracellular infections, their use is often restricted due to allergic reactions and other adverse side effects, especially in pregnant women and children. In this study, we proposed a potential beneficial role of DMI in controlling intracellular *Brucella* infection. In murine models, *Brucella* preferentially colonizes the spleen and liver, with the spleen typically showing higher bacterial loads. Although the reduction in liver bacterial load in DMI-treated mice did not reach statistical significance (*p* = 0.14), the observed decrease of 0.82 log_10_ CFU compared with the control group suggests a biologically relevant trend toward reduced bacterial persistence. The lack of significance may be partly due to the limited sample size (*n* = 6 per group) and inter-individual variability in bacterial colonization and immune responses. Additionally, organ-specific immune microenvironments may influence the magnitude of antimicrobial effects. Therefore, this trend may still indicate a meaningful effect of DMI treatment, which should be further validated in studies with larger cohorts or extended infection periods (30, 31). DMI has also been reported to inhibit infection caused by the obligate intracellular bacterium, *Anaplasma phagocytophilum* (19). The direct bactericidal effect of DMI against *Brucella* could contribute to reduced persistence of the pathogen in mice, besides potential positive effects on the host immunity against the infection.

DMI treatment also resulted in augmented CD4^+^ T cell populations and the production of several pro-inflammatory cytokines as observed *in vivo*. CD4^+^ T cells are crucial for *Brucella* clearance as they activate macrophages through IFNγ signaling (32). In the present study, an induced serum titer for IgG2a by the DMI-treated mice during *Brucella* infection was observed. This is known as a key antibody isotype in the acute or early stages of the immune response against *Brucella* infection in mice (33, 34). It is an indication of a Th1-mediated immune response, which is crucial in enhancing *Brucella* clearance and inhibiting bacterial replication in mice (35). Furthermore, increased production of serum cytokines and a chemokine was observed in DMI-treated mice, including TNFα, IL6, IL12, and MCP1 in the non-infected groups, suggesting an immunomodulatory effect of DMI. TNFα has been reported to be a key mediator in promoting cell-mediated resistance against *Brucella* infection and as a potent immunoregulatory cytokine produced by many cell types as part of the host defense against *Brucella* infection that contributes to the initiation, upregulation, and perpetuation of inflammatory response (36). IL6 has been reported to play an important role in priming the Th1 cell immune responses during *Brucella* infection and in activating bactericidal activity of macrophages (37). IL12 is known to be essential for controlling intracellular microbial infections and for stimulating both innate and adaptive immunity (38). MCP1 recruits monocytes and macrophages to the infection site, which can also aid in *Brucella* clearance but also in its chronic survival (39). With these, DMI properties could be further studied for its potential as a vaccine adjuvant. During *Brucella* infection, the DMI-treated mice displayed induced production of IL12, TNFα, IFNγ, IL6, and MCP1. These cytokines are known to play a major role in regulating immune response to *Brucella* infection as well as mediating the production of pro- and anti-inflammatory signals (40). IFNγ is considered the most important protective cytokine against intracellular pathogens (27). In addition, reduced IL10 serum level was observed in these *Brucella-*infected mice that received DMI treatment. *Brucella* induces IL10 production that favors persistence of the pathogen, and this plays a key role in evading host immunity, survival inside host cells, and establishing chronic infection (41–43).

In light of the findings from the present study, we provide evidence supporting the potential beneficial effects of DMI as a host cell-directed therapeutic for treating *B. abortus* infection. DMI exhibited bactericidal activity, reduced bacterial uptake and intracellular survival, and enhanced the expression of genes involved in controlling intracellular *Brucella*. Furthermore, DMI treatment demonstrated the potential to modulate immune responses against *Brucella* infection in animals.

## Data Availability

The raw data supporting the conclusions of this article will be made available by the authors, without undue reservation.

## Supplementary material

Supplementary Figure S1 Representative flow cytometry gating strategy for CD4⁺ and CD8⁺ T cells.
