# Supplementary material for: Desipramine treatment disrupts phagocytic and intracellular survival of Brucella abortus 544 in RAW 264.7 cells and promotes bacterial resistance with enhanced immune responses in ICR mice
Source: Front Vet Sci. 2026 May 13;13:1779164. doi: 10.3389/fvets.2026.1779164 (PMC13212344; doi:10.3389/fvets.2026.1779164)
Supplement: Supplementary Figure S1 — Representative flow cytometry gating strategy for CD4⁺ and CD8⁺ T cells. [file Image_1.pdf]

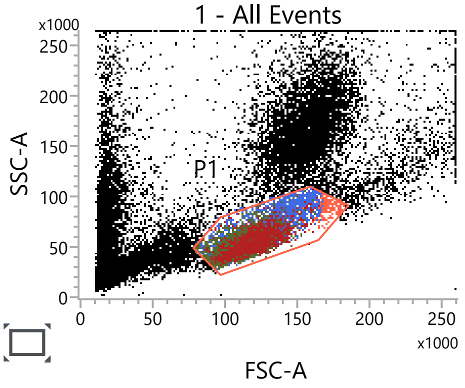

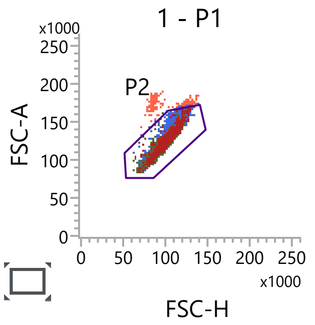

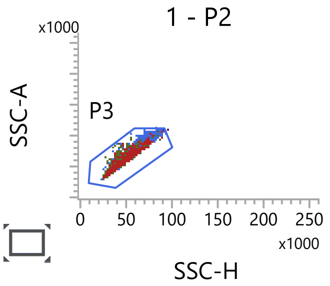

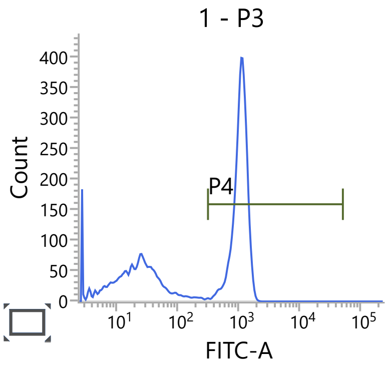

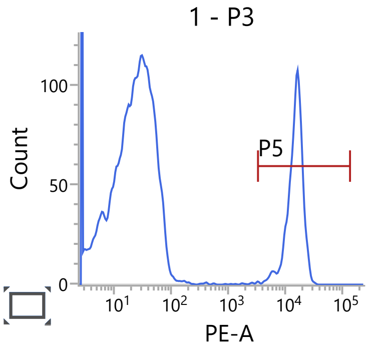

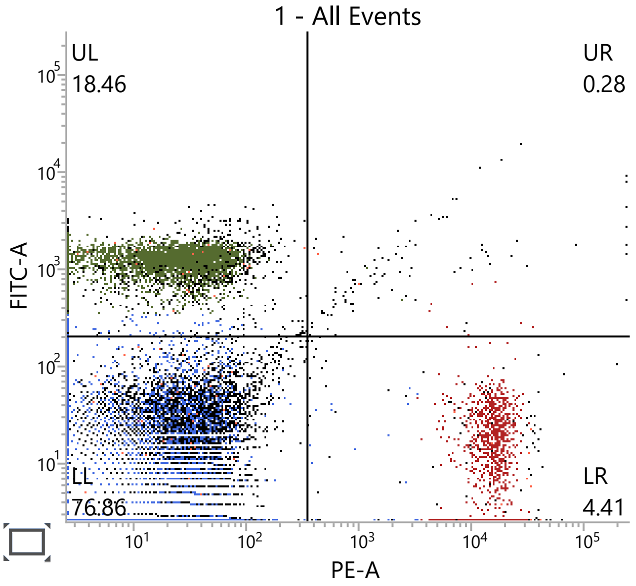

Statistics

Name	Events	% Parent	% Grandparent	% Total	FITC-A Mean	FITC-A Geo Mean	PE-A Mean	PE-A Geo Mean
1:P3	10,000	99.98	97.75	26.36	601	86	2,540	20
1:P4	4,963	49.63	49.62	13.08	1,194	1,153	14	6
1:P5	1,494	14.94	14.94	3.94	6	5	16,926	16,134
2:P3	10,000	99.98	96.42	25.68	569	139	2,294	22
2:P4	6,200	62.00	61.99	15.92	907	881	20	6
2:P5	1,625	16.25	16.25	4.17	5	4	14,022	13,420
3:P3	10,000	99.98	97.62	17.22	546	83	1,819	17
3:P4	4,845	48.45	48.44	8.34	1,106	1,064	10	5
3:P5	1,308	13.08	13.08	2.25	5	4	13,624	13,005
4:P3	10,000	99.98	97.14	18.86	617	156	1,670	19
4:P4	6,419	64.19	64.18	12.11	952	918	12	6
4:P5	1,518	15.18	15.18	2.86	7	5	10,898	10,308
5:P3	10,000	99.99	97.88	27.01	507	77	1,376	20
5:P4	4,862	48.62	48.62	13.13	1,025	994	11	7
5:P5	1,370	13.70	13.70	3.70	4	4	9,918	9,414
6:P3	10,000	99.99	98.43	33.69	589	91	3,155	39
6:P4	5,149	51.49	51.48	17.35	1,129	1,098	23	6
6:P5	2,384	23.84	23.84	8.03	7	5	13,178	12,644
7:P3	10,000	100.00	95.82	21.64	505	82	1,663	16
7:P4	4,492	44.92	44.92	9.72	1,085	1,035	16	6
7:P5	1,207	12.07	12.07	2.61	6	4	13,661	12,948
8:P3	10,000	99.99	97.84	17.34	591	102	1,976	21
8:P4	5,255	52.55	52.54	9.11	1,106	1,067	22	8
8:P5	1,342	13.42	13.42	2.33	9	5	14,591	13,674
9:P3	10,000	99.99	97.43	42.50	666	169	1,937	18
9:P4	5,946	59.46	59.45	25.27	1,095	1,057	17	6
9:P5	1,231	12.31	12.31	5.23	5	4	15,571	14,856
10:P3	10,000	99.98	98.01	33.13	666	124	2,276	18
10:P4	5,540	55.40	55.39	18.35	1,184	1,146	65	6
10:P5	1,382	13.82	13.82	4.58	7	4	16,179	15,526
11:P3	10,000	100.00	99.31	30.22	714	145	2,528	23
11:P4	5,883	58.83	58.83	17.78	1,202	1,167	35	6
11:P5	1,530	15.30	15.30	4.62	11	5	16,411	15,768
12:P3	10,000	100.00	97.79	43.53	597	112	3,017	34
12:P4	5,400	54.00	54.00	23.50	1,080	1,028	66	6
12:P5	2,160	21.60	21.60	9.40	7	5	13,732	12,805
13:P3	10,000	99.96	98.68	26.01	652	132	2,285	21
13:P4	5,543	55.43	55.41	14.42	1,153	1,112	74	6
13:P5	1,629	16.29	16.28	4.24	9	5	13,783	13,240
14:P3	10,000	99.99	98.58	32.97	833	266	1,654	16
14:P4	6,970	69.70	69.69	22.98	1,184	1,148	14	6
14:P5	1,275	12.75	12.75	4.20	9	5	12,716	12,112
15:P3	10,000	99.97	97.54	27.10	532	86	1,888	22
15:P4	5,069	50.69	50.67	13.74	1,031	988	21	6
15:P5	1,575	15.75	15.75	4.27	8	5	11,818	10,689
16:P3	10,000	99.91	97.18	12.66	453	80	1,051	18
16:P4	4,945	49.45	49.41	6.26	898	866	19	6
16:P5	1,165	11.65	11.64	1.48	9	5	8,589	7,889
17:P3	10,000	99.98	98.49	29.05	570	103	1,861	20
17:P4	4,969	49.69	49.68	14.44	1,120	1,083	27	6
17:P5	1,444	14.44	14.44	4.20	12	5	12,778	12,146
18:P3	10,000	99.99	97.80	18.13	522	102	1,628	22
18:P4	5,392	53.92	53.91	9.78	950	921	25	9
18:P5	1,256	12.56	12.56	2.28	8	5	12,525	11,863
19:P3	10,000	99.97	98.14	15.62	621	175	1,375	15
19:P4	6,641	66.41	66.39	10.38	924	892	52	5
19:P5	1,204	12.04	12.04	1.88	8	5	11,075	10,513
20:P3	10,000	99.95	97.45	36.62	669	178	1,865	19
20:P4	6,013	60.13	60.10	22.02	1,085	1,038	18	6
20:P5	1,330	13.30	13.29	4.87	10	5	13,770	13,089
21:P3	10,000	99.98	97.84	22.26	437	64	2,107	29
21:P4	4,128	41.28	41.27	9.19	1,019	974	46	7
21:P5	1,808	18.08	18.08	4.02	11	6	11,475	10,563
22:P3	10,000	99.95	98.33	21.08	540	88	1,707	17
22:P4	4,842	48.42	48.40	10.21	1,089	1,051	67	6
22:P5	1,218	12.18	12.17	2.57	8	5	13,671	12,823
23:P3	10,000	99.99	99.14	37.62	665	135	2,605	23
23:P4	5,477	54.77	54.76	20.60	1,190	1,149	68	6
23:P5	1,559	15.59	15.59	5.86	10	5	16,421	15,854
24:P3	10,000	99.99	98.28	36.25	611	117	3,756	42
24:P4	5,506	55.06	55.05	19.96	1,087	1,037	23	6
24:P5	2,403	24.03	24.03	8.71	8	5	15,579	15,055
25:P3	10,000	99.98	98.53	47.55	549	102	4,044	45
25:P4	5,111	51.11	51.10	24.30	1,044	996	42	6
25:P5	2,473	24.73	24.73	11.76	11	5	16,293	15,695
26:P3	10,000	100.00	98.76	44.09	611	114	1,970	18
26:P4	5,393	53.93	53.93	23.78	1,115	1,075	69	6
26:P5	1,407	14.07	14.07	6.20	9	5	13,673	12,837
27:P3	10,000	99.99	98.78	25.51	777	193	3,367	26
27:P4	6,220	62.20	62.19	15.87	1,230	1,185	64	6
27:P5	1,773	17.73	17.73	4.52	10	5	18,771	18,070
